# Comparative sensory and proximate evaluation of spontaneously fermenting kunu-zaki made from germinated and ungerminated composite cereal grains

**DOI:** 10.1002/fsn3.45

**Published:** 2013-06-18

**Authors:** Solakunmi O Oluwajoba, Felix A Akinyosoye, Olusegun V Oyetayo

**Affiliations:** Department of Microbiology, School of Science, Federal University of TechnologyAkure, Ondo State, Nigeria

**Keywords:** Composite, germinated, kunu-zaki, noncomposite, sensory evaluation, ungerminated

## Abstract

This study evaluated the sensory properties, proximate composition, and overall consumer acceptability of kunu-zaki using germinated and ungerminated *Sorghum bicolor* (sorghum), *Pennisetum americanum* (millet), and *Digitaria exilis* (acha) cereal grains. The three cereal grains were used in nongerminated and germinated composite and noncomposite proportions coded A (Acha), S (Sorghum), M (Millet), AS (Acha–Sorghum), AM (Acha–Millet), SM (Sorghum–Millet), ASG (Acha–Sorghum Germinated), AMG (Acha–Millet Germinated), and SMG (Sorghum–Millet Germinated). Proximate analysis determined the moisture content, ash, crude fiber, fat, and crude protein content of the fermented grains. The 9-point hedonic scale was used to judge the sensory parameters of taste, color, and aroma. The paired comparison test was used to judge consumer preference between kunu-zaki made from germinated grains and the ungerminated counterpart. Scores were statistically analyzed using the Kruskal–Wallis test in the SPSS analytical software package. Panelists ranked the ASG-coded drink highest in terms of taste and aroma, the AMG-coded drink highest in terms of color. SM ranked least in terms of taste; SMG ranked least in terms of aroma; and AM ranked the least in terms of color. Preference for each parameter was significantly different (*P* < 0.001). Panelists ranked overall preference for the drinks from the most liked to the least liked in the order ASG>AMG>A>AS>S>M>SMG>AM>SM. The overall preference for the drinks was also significantly different (*P* < 0.001). Panelists pairing both ungerminated drinks with the germinated drinks ranked the ungerminated drink AS as most preferred in terms of taste, color, and aroma above its germinated counterpart ASG with preference not significantly dependent on the parameters (*P* = 0.065 > 0.05). Ungerminated AM was also preferred above the germinated counterpart AMG in terms of taste, color, and aroma with preference not significantly dependent on parameters (*P* = 0.055 > 0.05). However, panelists showed preference for the taste and aroma of the germinated drink SMG but more preference for the color of the ungerminated drink SM with preference significantly dependent on the parameters (*P* = 0.028 < 0.05). Crude fiber values were higher – 11.3%, 13.1%, and 17.37% for SMG, AMG and ASG, respectively. Germination increased %Fat values slightly but the %Ash was relatively stable in both germinated and ungerminated drinks. Addition of germinated acha cereal grains to either sorghum or millet prior to fermentation offers desirable sensory and nutritional quality attributes in kunu-zaki.

## Introduction

For many years, the Nigerian soft drink industry has been heavily dependent on imported raw materials. In order to conserve foreign exchange, emphasis is now on the development of indigenous beverages and the country's attention has begun to shift toward the local sourcing of raw materials for economic development (Obadina et al. 2008). Kunu-zaki is an indigenous fermented beverage made from unsprouted cereal grains (Adeyemi and Umar 1994). The drink has its origin in the northern parts of Nigeria but is now popular in almost all the states in Nigeria (Gaffa and Ayo 2002). Cereal grains form a major source of dietary nutrients for all people, particularly those in the developing countries (Slavin 2010). However, the nutritional quality of cereal grains and sensory properties of their products are inferior due to lower protein content, deficiency of certain essential amino acids, lower protein and starch availabilities, presence of certain antinutrients, and the coarse nature of the grains (Kahlon 2009). Germination of cereal seeds has been reported as an effective processing treatment to improve the nutritional quality of cereals (Amadou et al. 2011). Sprouting of grains for a limited period causes increased activities of hydrolytic enzymes, improvement in the contents of certain essential amino acids, total sugars, and B-group vitamins, and a decrease in dry matter, starch, and antinutrients (Akinhanmi et al. 2008). The digestibility of storage proteins and starch is improved due to their partial hydrolysis during sprouting (Inyang and Zakari 2008). The magnitude of the nutritional improvement is, however, influenced by the type of cereal, seed quality, and sprouting conditions (Kahlon 2009). The consumption of sprouted cereals is gradually becoming popular in various parts of the world (Slavin 2010). Kunu-zaki (sweet kunu) is a cheap traditional nonalcoholic fermented beverage widely consumed especially during the dry season (Adeyemi and Umar 1994). Kunu-zaki processing is mostly done by women using simple household equipment and utensils. Depending on cereal availability, the unsprouted cereal grains used for kunu-zaki processing are sorghum, maize, millet, guinea corn, or rice in mostly noncomposite proportions. Even though kunu-zaki is popular and fast becoming a household technology in the country, the cereal grains used are selected randomly and diversely. For instance, some women use millet only or sorghum only, while others use only maize. Some also use sorghum with rice, while others use millet with sorghum. The most popular cereals used are sorghum and millet. When kunu-zaki is made from sorghum, the final product is a light-brown liquid. When made from millet, the resulting liquid product is milky white. Investigative studies on comparative sensory properties or consumer preferences of the drink when produced from these different grains are nonexistent. Furthermore, few scientists (Nzelibe et al. 2000; Daramola et al. 2008; Inyang and Zakari 2008) have investigated the effect of some selected malted cereals on properties of kunu-zaki but the sensory properties of each resulting drink were examined only in isolation. Studies have not yet been conducted to compare either the sensory properties and determine the overall acceptability of kunu-zaki when made from sprouted composite cereals, unsprouted composite cereals, composite cereal mixes, or noncomposite cereal proportions. Kunu-zaki is not conventionally produced using sprouted cereals and acha is not a cereal commonly used in kunu-zaki production in spite of its established nutritional advantage. This study therefore sought to compare the sensory properties, the resulting proximate composition, and overall consumer acceptability of kunu-zaki when produced from sprouted and unsprouted cereals in either composite or noncomposite proportions. The conventional grains used in the production of kunu-zaki (sorghum and millet) were used with acha being introduced into the kunu-zaki processing as a composite complement.

## Materials and Methods

### Laboratory production of kunun-zaki

**Figure 1:**
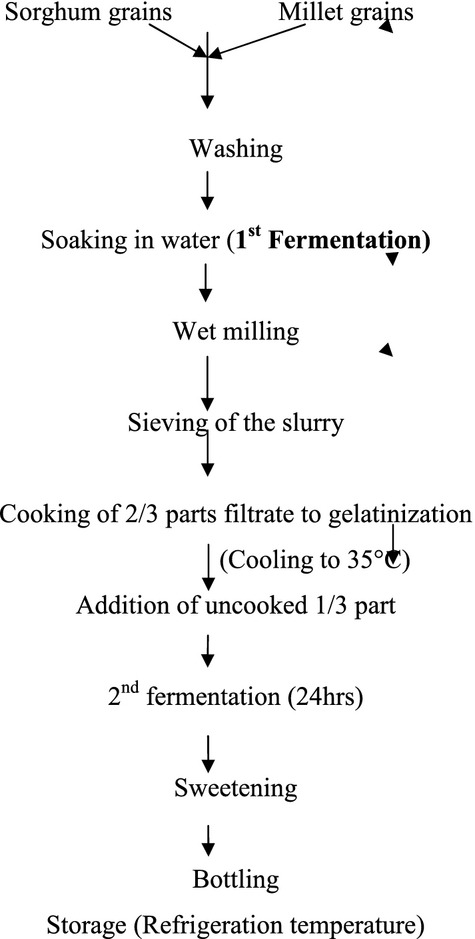
Flow diagram for the traditional processing of kunu-zaki from composite grains.

Sorghum, *Sorghum bicolor*, millet, *Pennisetum americanum*, and Hungry rice (locally known as fonio or acha), *Digitaria exilis* grains were obtained from the Nigeria Cereal Research Institute in Ibadan. The grains were cleaned, weighed, and washed before steeping in distilled water. Two hundred grams of cereal grains was used for the kunun-zaki production. A control experiment was set up with distilled water without the grains. For the kunun-zaki made from composite grains (Fig. 1), an equal weight of grains was used for each part. The laboratory production method followed the traditional process of kunun-zaki fermentation.

### Composite cereals

The composite grains were used in the ratio of 50:50 and coded as follows: AS, Acha/Sorghum ungerminated composite grains; AM, Acha/Millet ungerminated composite grains; SM, Sorghum/Millet ungerminated composite grains; ASG, Acha/Sorghum Germinated composite grains; AMG, Acha/Millet Germinated composite grains; SMG, Sorghum/Millet Germinated composite grains.

### Germination of cereal grains

Two hundred grams of cereal grains was rinsed in distilled water and drained. Steeping was carried out at a temperature of 48°C to a moisture content of about 42–45%. Water was drained and germination carried out by spreading the steeped grains on a tray, in a room at temperature of 28 ± 2°C. Seeds were sprayed intermittently with water. The germinated grains were recovered when the radical was about 1.5 mm in length.

### Proximate composition

Estimations were made of nitrogen (as an index of crude protein), water, fat, ash, and crude fiber. When the total was subtracted from 100%, the difference was termed carbohydrate by difference. Determination of the moisture content, ash, and crude fat followed the method of AOAC (1990). Crude fiber determination followed the method of Pearson (1981). Estimation of nitrogen content was by the Kjeldahl method multiplied by 6.25, the nitrogen-protein factor to convert to crude protein.

#### Moisture content

The moisture content was determined using procedure described by AOAC (1990). The moisture content of each sample was determined by weighing 5 g of the sample into a aluminum moisture can. The sample was then dried to constant weight at 105 ± 2°C.


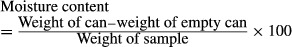


#### Crude protein and fat content

The protein and fat content of the samples were determined (AOAC 1990).

#### Ash content

Two grams of samples was weighed in well incinerated crucibles and then ashed in a muffle furnace at 600°C for 3 h. The ash content was calculated as


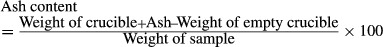


#### Crude fiber

Two grams of the sample was transferred into a 1 L conical flask. One hundred milliliters of sulfuric acid (0.255 mol/L) was heated to boiling and then introduced into the conical flask containing the sample. The contents were then boiled for 30 min, ensuring that the level of the acid was maintained by the addition of distilled water. After 30 min, the contents were then filtered through a muslin cloth held in a funnel. The residue was rinsed thoroughly until its washing was no longer acidic to litmus. The residue was then transferred into a conical flask. One hundred milliliters of sodium hydroxide (0.313 mol/L) was then brought to boil and then introduced into the conical flask containing the sample. The contents were then boiled for 30 min, ensuring that the level of the acid was maintained by the addition of distilled water. After 30 min, the contents were then filtered through a muslin cloth held in a funnel. The residue was rinsed thoroughly until its washing was no longer alkali. The residue was then introduced into an already dried crucible and ashed at 600°C ± 200°C.


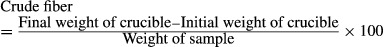


### Sensory evaluation

The 9-point hedonic scale assessment and the paired comparison tests were used as described by Larmond (1977). A total of 87 untrained panelists from a cross-section of students and staff of the Department of Food Science from the Federal University of Technology, Akure, Ondo State and the Bells University of Technology, Ota, Ogun State communities were selected based on their familiarity with kunu-zaki beverage. The panelists scored coded drinks in terms of degree of liking to taste, color, and aroma. The 9-point hedonic scale used by the panelists for the evaluation ranged from 1 to 9 representing “extremely dislike” to “extremely like”. The coded samples were served in clean, transparent cups at room temperature 25°C. The panelists used booths that were illuminated with fluorescent light and the coded drinks were tasted one at a time. Panelists also scored preference between the coded drinks made from germinated or ungerminated grains. Water was given to each panelist for oral rinsing in between tasting of the samples. One sensory attribute was tested for at each sitting (Tables 1–8).

**Table 1:** Kruskal–Wallis test (parameter = taste)

	Ranks		
	
	Types of drink	*N*	Mean rank
Response	SM	225	734.84
	A	222	1098.89
	AS	225	1031.60
	M	225	1021.58
	SMG	225	881.72
	ASG	225	1505.30
	AM	225	818.12
	AMG	225	1150.04
	S	225	862.58
	Total	2022	

**Table 2:** Taste test statistics (parameter = taste)^1^,^2^

	Response
Chi-square	288.127
df	8
Asymp. significance	0.000

1 Kruskal–Wallis test.

2 Grouping variable: types of drink.

**Table 3:** Kruskal–Wallis test (parameter = aroma)

	Ranks		
	
	Types of drink	*N*	Mean rank
Response	SM	225	758.30
	A	216	994.44
	AS	171	796.05
	M	225	914.54
	SMG	225	735.44
	ASG	225	1545.62
	AM	225	768.92
	AMG	225	1205.78
	S	225	1070.42
	Total	1962	

**Table 4:** Aroma test statistics (parameter = aroma)^1^,^2^

	Response
Chi-square	404.054
df	8
Asymp. significance	0.000

1 Kruskal–Wallis test.

2 Grouping variable: types of drink.

**Table 5:** Kruskal–Wallis test (parameter = color)

	Ranks		
	
	Types of drink	*N*	Mean rank
Response	SM	225	644.54
	A	225	1191.20
	AS	135	1078.10
	M	225	676.22
	SMG	225	890.60
	ASG	225	1142.96
	AM	225	619.88
	AMG	225	1667.84
	S	225	844.70
	Total	1935	

**Table 6:** Color test statistics (parameter = color)^1^,^2^

	Response
Chi-square	677.701
df	8
Asymp. significance	0.000

1 Kruskal–Wallis test.

2 Grouping variable: types of drink.

**Table 7:** Kruskal–Wallis test: overall

	Ranks		
	
	Types of drink	*N*	Mean rank
Response	SM	675	2130.54
	A	663	3298.15
	AS	531	2894.81
	M	675	2604.80
	SMG	675	2527.54
	ASG	675	4127.76
	AM	675	2222.36
	AMG	675	4072.94
	S	675	2753.20
	Total	5919	

**Table 8:** Test statistics^1^,^2^

	Response
Chi-square	1022.658
df	8
Asymp. significance	0.000

1 Kruskal–Wallis test.

2 Grouping variable: types of drink.

The scores were statistically analyzed using the Kruskal–Wallis test in the SPSS analytical software package (IBM SPSS Inc., Chicago, IL).

## Results and Discussion

**Figure 2:**
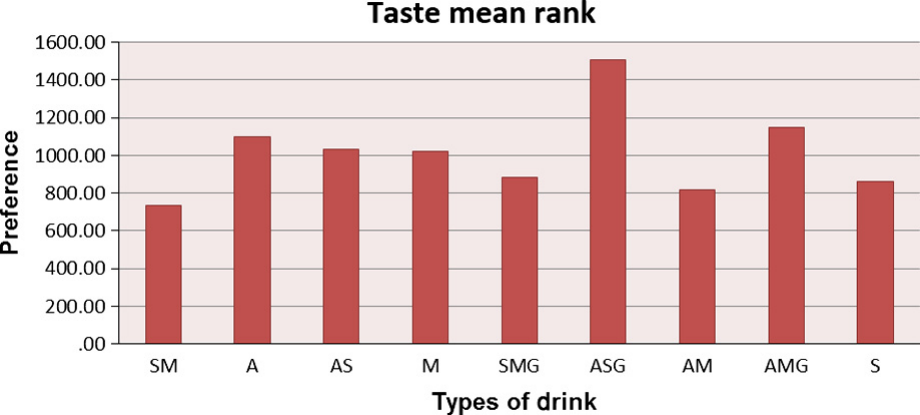
Panelists TASTE preference for kunu-zaki drinks made from germinated and ungerminated composite cereals. The results above show that ASG ranked highest, hence the most liked of all in terms of taste while SM was the least liked. The test shows that preference for the drinks is significantly different (*P* < 0.001).

**Figure 3:**
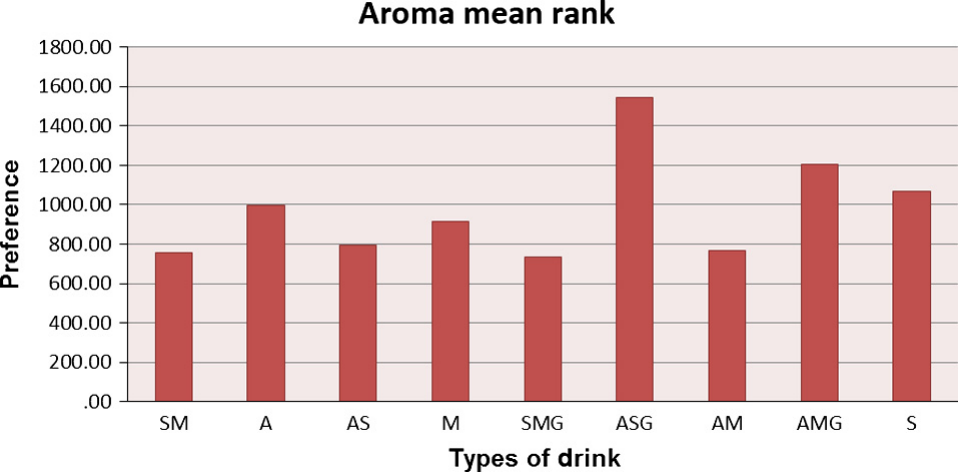
Panelists AROMA preference for kunu-zaki drinks made from germinated and ungerminated composite cereals. The results above show that ASG ranked highest, hence the most liked of all in terms of aroma while SMG was the least liked. The test shows that preference for the drinks is significantly different too (*P* < 0.001).

**Figure 4:**
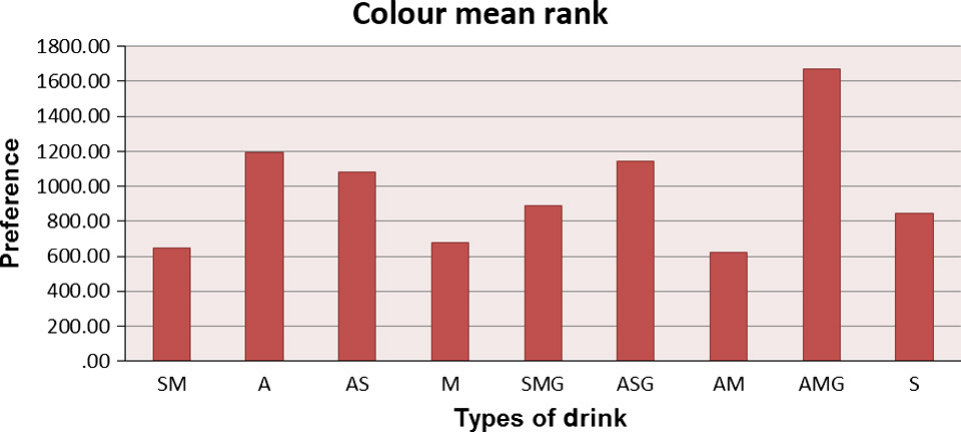
Panelists COLOR preference for kunu-zaki drinks from germinated and ungerminated cereals. The results above show that AMG ranked highest, hence the most liked of all in terms of color while AM was the least liked. The test shows that preference for the drinks is significantly different (*P* < 0.001).

**Figure 5:**
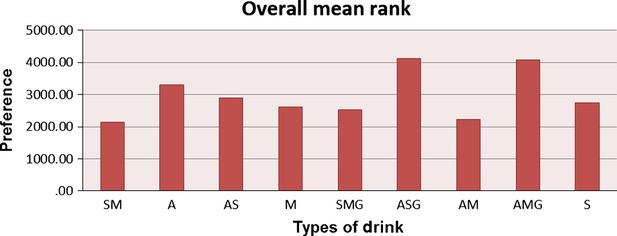
Panelists OVERALL preference for kunu-zaki drinks from germinated and nongerminated cereals. The results above show that ASG ranked highest, hence the most liked of all while SM was the least liked. The test shows that preference for the drinks is significantly different (*P* < 0.001). Ranking from the most liked: ASG>AMG>A>AS>S>M>SMG>AM>SM.

**Figure 6:**
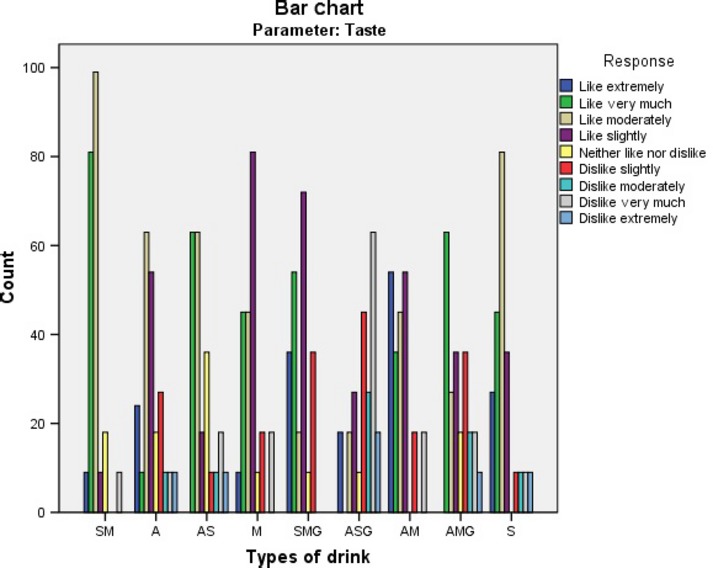
Paired comparison between nongerminated composite grains SM (Sorghum–Millet) and the germinated counterpart SMG (Sorghum–Millet Germinated).

**Figure 7:**
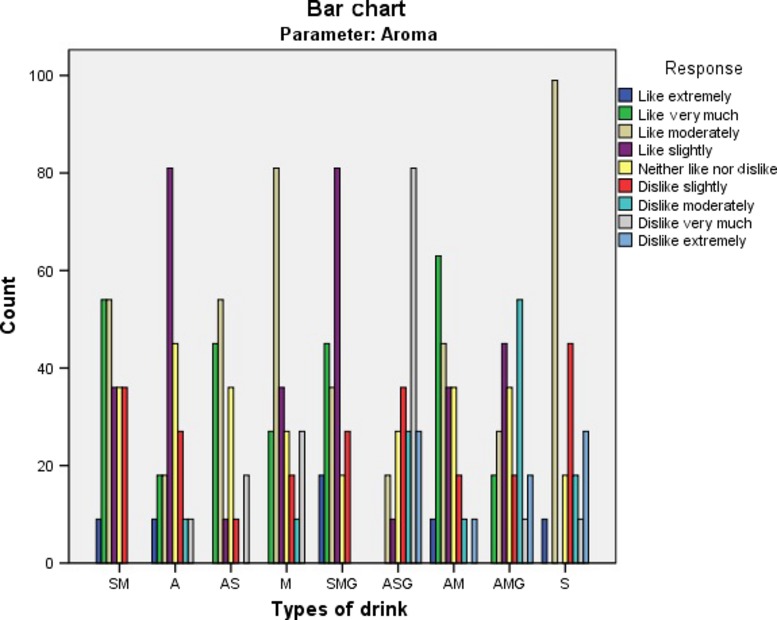
Paired comparison between nongerminated composite grains AM (Acha–Millet) and the germinated counterpart AMG (Acha–Millet Germinated).

**Figure 8:**
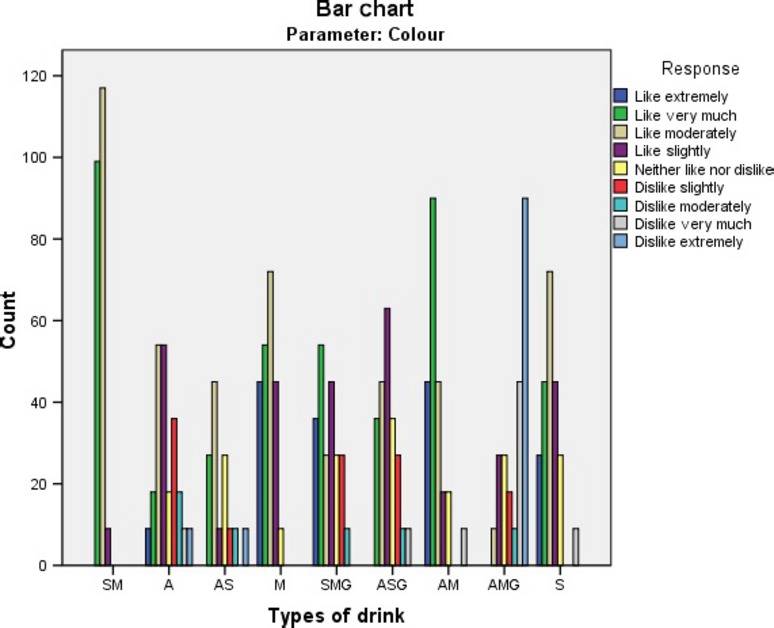
Panelists' hedonic assessment on TASTE.

**Figure 9:**
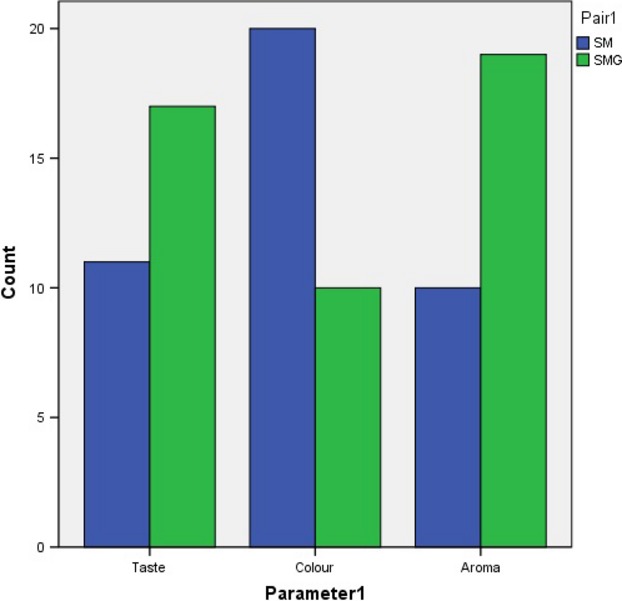
Panelists' hedonic assessment on AROMA.

**Figure 10:**
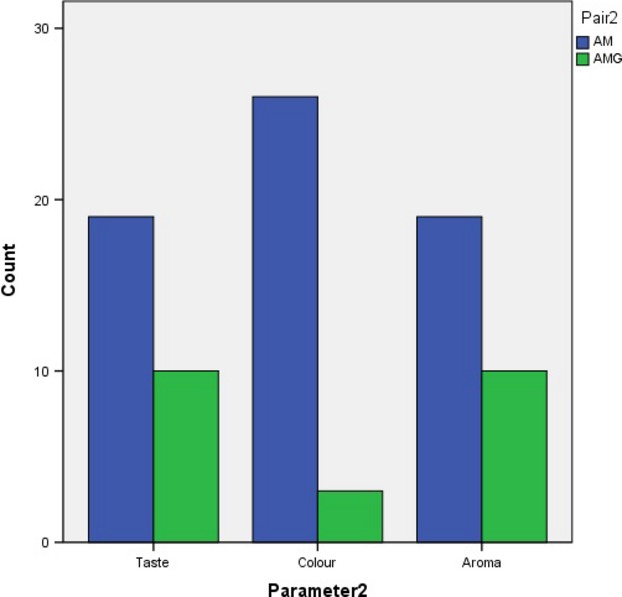
Panelists' hedonic assessment on COLOR.

**Figure 11:**
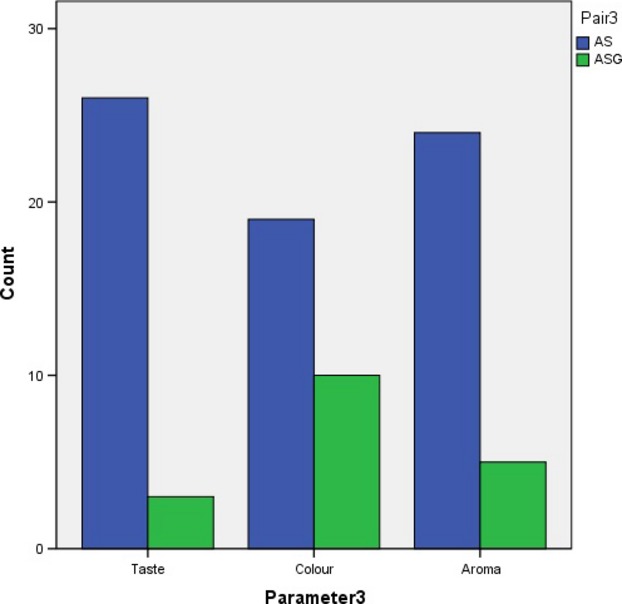
Paired comparison between nongerminated composite grains AS (Acha–Sorghum) and the germinated counterpart ASG (Acha–Sorghum Germinated).

Figure 2 shows that ASG (Acha–Sorghum Germinated) was ranked highest by panelists, hence the most liked of all in terms of taste. SM (Sorghum–Millet) had the least liked taste. The preference for the taste of the drinks was significantly different (*P* < 0.001). Figure 3 similarly shows that ASG (Acha–Sorghum Germinated) was again ranked highest by panelists in terms of aroma while the aroma of SMG (Sorghum–Millet Germinated) was the least liked. The preference for the drinks was significantly different too (*P* < 0.001). Figure 4 shows that AMG (Acha–Millet Germinated) was, however, ranked highest by panelists in terms of color while AM (Acha–Millet) was the least liked. The preference for the COLOR of the drinks was significantly different (*P* < 0.001). The results from Figure 5 show that ASG (Acha–Sorghum Germinated) was given the overall highest rating by panelists, hence the most liked of all while SM (Sorghum–Millet) was the least liked. The preference for the drinks on the overall was also significantly different (*P* < 0.001). Ranking from the most liked ASG>AMG>A>AS>S>M>SMG>AM>SM. From the overall ranking, it is easy to see that kunu-zaki drinks made from germinated grains of sorghum and acha or millet and acha grains were rated high by panelists. This is an indication of the acceptability of acha cereal grains for production of the fermented beverage in composite mixes. It is interesting to note that kunu-zaki made from acha cereal alone was rated quite poorly by panelists. These results agree with earlier preliminary studies by Gaffa and Jideani (2001) and Gaffa et al. (2001, 2002a,b, 2004). Jideani (1999) had highlighted the possible technological uses of *D. exilis*. Acha has become an attractive focus in recent times and its research has covered many areas (Jideani and Akingbala 1993; Jideani et al. 1994a,b; Ayo et al. 2008). In reviews by Jideani (1999), Jideani and Jideani (2011), and some other workers (Balde et al. 2008), it was reported that acha helps diabetic patients. These previous discoveries are now complemented by the results of sensory studies from this research work. Jideani (1993) had earlier demonstrated the production of kunu-zaki from acha grains alone with the resulting drink labeled “kunu-acha.” From the paired comparisons, Figure 6 shows that when kunu-zaki drinks made from germinated and nongerminated grains were compared simultaneously, panelists showed a higher preference to the taste and aroma of SMG (Sorghum–Millet Germinated) than the nongerminated counterpart SM (Sorghum–Millet). Various studies (Mbith-Mwikya et al. 2000; Makokha et al. 2002; Wadikar 2006; Inyang and Zakari 2008) have shown that germination followed by fermentation of millets and sorghum significantly reduce the amount of antinutrients (tannin and phytate) leading to effective starch and protein hydrolysis and increased mineral bioavailability. Even though these works did not compare sensory properties, the results of this study have now also established sensory preference in addition to the nutritional advantage. Figure 7 shows that for the three parameters of color, taste, and aroma, panelists rated the ungerminated cereal drink acha–millet higher than the germinated counterpart AMG. This agrees with the work of Ayo (2004). Even though the work of Ayo (2004) did not include germination, sensory properties of acha inclusion in the production of kunu-zaki were rated very high by panelists. These results are also comparable to that of Inyang and Zakari (2008) and Nzelibe et al. (2000). All the drinks made from fermented noncomposite cereal grains ranked lower than all the drinks made from fermented composite grains. Out of all the kunu-zaki drinks made from noncomposite cereals, the acha-based noncomposite cereal drink was still rated higher. In their overall judgments, panelists preferred drinks made from cereal grains which contained acha in the composite mix. Akoma et al. (2012) also found that the addition of malted rice to millet was preferred organoleptically. Badau (2007) also reported that malting improved the taste of kunu-zaki formulations using pearl millet alone. Tables 9–14 explain the significant test results of the kunu-zaki drinks when panelists assessed each of the drinks for taste, color, and aroma on a 9-point hedonic scale. The bar chart of the panelists assessments explains more clearly the pattern of scoring by the panelists (Figs. 8–11). The inclusion of acha in composite proportion to either sorghum or millet also had a considerable impact on composite proximate composition (Tables 15–20). Tables 21 and 23 show the proximate composition of both germinated and ungerminated acha–millet grains revealing higher protein and crude fiber contents relative to the other composite grains. These differences were, however, not significant (Tables 22 and 24). Germination did have an increased effect on the protein content as the germinated counterpart of the grains showed an increase. These results complement the findings of Litchenwainer et al. (1979). In the present study, percentage values of protein in kunu-zaki from composite mix ranged between 13.9% and 18.54%, fat values were between 0.43% and 4.1%, ash content ranged from 1.2% to 4%, crude fiber values were between 8.0% and 17.73%, and carbohydrates ranged between 70.4% and 80.6%. Litchenwainer et al. (1979) had reported that kunu-zaki processed from sorghum alone contains 11.6% protein, 3.3% fat, 1.9% ash, and 76.8% carbohydrate. This study therefore demonstrates that composite cereal blends exhibit a better nutritional advantage over the noncomposite cereal blends in kunu-zaki production.

**Table 9:** Panelists' hedonic scale assessment (parameter = taste)

			Response									
		
Types of drink × Response cross-tabulation			Like extremely	Like very much	Like moderately	Like slightly	Neither like nor dislike	Dislike slightly	Dislike moderately	Dislike very much	Dislike extremely	Total
Types of drink	SM	Count	9	81	99	9	18	0	0	9	0	225
		% within response	5.1%	20.5%	21.6%	2.3%	15.4%	0.0%	0.0%	5.6%	0.0%	11.1%
	A	Count	24	9	63	54	18	27	9	9	9	222
		% within response	13.6%	2.3%	13.7%	14.0%	15.4%	13.6%	12.5%	5.6%	16.7%	11.0%
	AS	Count	0	63	63	18	36	9	9	18	9	225
		% within response	0.0%	15.9%	13.7%	4.7%	30.8%	4.5%	12.5%	11.1%	16.7%	11.1%
	M	Count	9	45	45	81	9	18	0	18	0	225
		% within response	5.1%	11.4%	9.8%	20.9%	7.7%	9.1%	0.0%	11.1%	0.0%	11.1%
	SMG	Count	36	54	18	72	9	36	0	0	0	225
		% within response	20.3%	13.6%	3.9%	18.6%	7.7%	18.2%	0.0%	0.0%	0.0%	11.1%
	ASG	Count	18	0	18	27	9	45	27	63	18	225
		% within response	10.2%	0.0%	3.9%	7.0%	7.7%	22.7%	37.5%	38.9%	33.3%	11.1%
	AM	Count	54	36	45	54	0	18	0	18	0	225
		% within response	30.5%	9.1%	9.8%	14.0%	0.0%	9.1%	0.0%	11.1%	0.0%	11.1%
	AMG	Count	0	63	27	36	18	36	18	18	9	225
		% within response	0.0%	15.9%	5.9%	9.3%	15.4%	18.2%	25.0%	11.1%	16.7%	11.1%
	S	Count	27	45	81	36	0	9	9	9	9	225
		% within response	15.3%	11.4%	17.6%	9.3%	0.0%	4.5%	12.5%	5.6%	16.7%	11.1%
Total		Count	177	396	459	387	117	198	72	162	54	2022
	% within response	100.0%	100.0%	100.0%	100.0%	100.0%	100.0%	100.0%	100.0%	100.0%	100.0%

**Table 10:** Hedonic assessment for taste chi-square tests (parameter = taste)

	Value	df	Asymp. significance (2-sided)
Pearson chi-square	928.822^1^	64	0.000
Likelihood ratio	1042.638	64	0.000
Linear-by-linear association	10.800	1	0.001
Number of valid cases	2022		

1 0 cells (0.0%) have expected count less than 5. The minimum expected count is 5.93.

**Table 11:** Panelists hedonic assessment (parameter = aroma)

			Response									Total
	
Types of drink × Response cross-tabulation			Like extremely	Like very much	Like moderately	Like slightly	Neither like nor dislike	Dislike slightly	Dislike moderately	Dislike very much	Dislike extremely	
Types of drink	SM	Count	9	54	54	36	36	36	0	0	0	225
		% within response	16.7%	20.0%	12.5%	10.8%	12.9%	15.4%	0.0%	0.0%	0.0%	11.5%
	A	Count	9	18	18	81	45	27	9	9	0	216
		% within response	16.7%	6.7%	4.2%	24.3%	16.1%	11.5%	7.1%	5.9%	0.0%	11.0%
	AS	Count	0	45	54	9	36	9	0	18	0	171
		% within response	0.0%	16.7%	12.5%	2.7%	12.9%	3.8%	0.0%	11.8%	0.0%	8.7%
	M	Count	0	27	81	36	27	18	9	27	0	225
		% within response	0.0%	10.0%	18.8%	10.8%	9.7%	7.7%	7.1%	17.6%	0.0%	11.5%
	SMG	Count	18	45	36	81	18	27	0	0	0	225
		% within response	33.3%	16.7%	8.3%	24.3%	6.5%	11.5%	0.0%	0.0%	0.0%	11.5%
	ASG	Count	0	0	18	9	27	36	27	81	27	225
		% within response	0.0%	0.0%	4.2%	2.7%	9.7%	15.4%	21.4%	52.9%	33.3%	11.5%
	AM	Count	9	63	45	36	36	18	9	0	9	225
		% within response	16.7%	23.3%	10.4%	10.8%	12.9%	7.7%	7.1%	0.0%	11.1%	11.5%
	AMG	Count	0	18	27	45	36	18	54	9	18	225
		% within response	0.0%	6.7%	6.3%	13.5%	12.9%	7.7%	42.9%	5.9%	22.2%	11.5%
	S	Count	9	0	99	0	18	45	18	9	27	225
		% within response	16.7%	0.0%	22.9%	0.0%	6.5%	19.2%	14.3%	5.9%	33.3%	11.5%
Total		Count	54	270	432	333	279	234	126	153	81	1962
	% within response	100.0%	100.0%	100.0%	100.0%	100.0%	100.0%	100.0%	100.0%	100.0%	100.0%

**Table 12:** Hedonic assessment for aroma chi-square tests (parameter = aroma)

	Value	df	Asymp. significance (2-sided)
Pearson chi-square	1154.496^1^	64	.000
Likelihood ratio	1203.408	64	.000
Linear-by-linear association	87.965	1	.000
Number of valid cases	1962		

1 1 cell (1.2%) has expected count less than 5. The minimum expected count is 4.71.

**Table 13:** Panelists hedonic assessment (parameter = color)

			Response									Total
	
Types of drink × Response cross-tabulation			Like extremely	Like very much	Like moderately	Like slightly	Neither like nor dislike	Dislike slightly	Dislike moderately	Dislike very much	Dislike extremely	
Types of drink	SM	Count	0	99	117	9	0	0	0	0	0	225
		% within response	0.0%	23.4%	24.1%	2.9%	0.0%	0.0%	0.0%	0.0%	0.0%	11.6%
	A	Count	9	18	54	54	18	36	18	9	9	225
		% within response	5.6%	4.3%	11.1%	17.1%	9.5%	30.8%	33.3%	11.1%	8.3%	11.6%
	AS	Count	0	27	45	9	27	9	9	0	9	135
		% within response	0.0%	6.4%	9.3%	2.9%	14.3%	7.7%	16.7%	0.0%	8.3%	7.0%
	M	Count	45	54	72	45	9	0	0	0	0	225
		% within response	27.8%	12.8%	14.8%	14.3%	4.8%	0.0%	0.0%	0.0%	0.0%	11.6%
	SMG	Count	36	54	27	45	27	27	9	0	0	225
		% within response	22.2%	12.8%	5.6%	14.3%	14.3%	23.1%	16.7%	0.0%	0.0%	11.6%
	ASG	Count	0	36	45	63	36	27	9	9	0	225
		% within response	0.0%	8.5%	9.3%	20.0%	19.0%	23.1%	16.7%	11.1%	0.0%	11.6%
	AM	Count	45	90	45	18	18	0	0	9	0	225
		% within response	27.8%	21.3%	9.3%	5.7%	9.5%	0.0%	0.0%	11.1%	0.0%	11.6%
	AMG	Count	0	0	9	27	27	18	9	45	90	225
		% within response	0.0%	0.0%	1.9%	8.6%	14.3%	15.4%	16.7%	55.6%	83.3%	11.6%
	S	Count	27	45	72	45	27	0	0	9	0	225
		% within response	16.7%	10.6%	14.8%	14.3%	14.3%	0.0%	0.0%	11.1%	0.0%	11.6%
Total		Count	162	423	486	315	189	117	54	81	108	1935
		% within response	100.0%	100.0%	100.0%	100.0%	100.0%	100.0%	100.0%	100.0%	100.0%	100.0%

**Table 14:** Hedonic assessment for color chi-square tests (parameter = color)

	Value	df	Asymp. significance (2-sided)
Pearson chi-square	1491.132^1^	64	0.000
Likelihood ratio	1429.881	64	0.000
Linear-by-linear association	73.059	1	0.000
Number of valid cases	1935		

1 1 cell (1.2%) has expected count less than 5. The minimum expected count is 3.77.

**Table 15:** Paired comparison and chi-square test scores between SM and SMG

			Taste	Color	Aroma	Total
Pair1	SM	Count	11	20	10	41
		% within Parameter1	39.3%	66.7%	34.5%	47.1%
	SMG	Count	17	10	19	46
		% within Parameter1	60.7%	33.3%	65.5%	52.9%
Total		Count	28	30	29	87
		% within Parameter1	100.0%	100.0%	100.0%	100.0%

**Table 16:** Chi-square tests

	Value	df	Asymp. significance (2-sided)
Pearson chi-square	7.148^1^	2	0.028
Likelihood ratio	7.246	2	0.027
Linear-by-linear association	0.151	1	0.698
No of valid cases	87		

The chi-square table shows that preference was significantly dependent on the parameters (*P* = 0.028 < 0.05).

1 0 cells (0.0%) have expected count less than 5. The minimum expected count is 13.20.

**Table 17:** Paired comparison and chi square test scores between AM and AMG

			Taste	Color	Aroma	Total
Pair2	AM	Count	19	26	19	64
		% within Parameter2	65.5%	89.7%	65.5%	73.6%
	AMG	Count	10	3	10	23
		% within Parameter2	34.5%	10.3%	34.5%	26.4%
Total		Count	29	29	29	87
		% within Parameter2	100.0%	100.0%	100.0%	100.0%

**Table 18:** Chi-square tests

	Value	df	Asymp. significance (2-sided)
Pearson chi-square	5.792^1^	2	0.055
Likelihood ratio	6.482	2	0.039
Linear-by-linear association	0.000	1	1.000
No. of valid cases	87		

The chi-square table above shows that preference was not significantly dependent on parameters (*P* = 0.055 > 0.05).

1 10 cells (0.0%) have expected count less than 5. The minimum expected count is 7.67.

**Table 19:** Paired comparison and chi-square test scores between AS and ASG

			Taste	Color	Aroma	Total
Pair3	AS	Count	26	19	24	69
		% within Parameter3	89.7%	65.5%	82.8%	79.3%
	ASG	Count	3	10	5	18
		% within Parameter3	10.3%	34.5%	17.2%	20.7%
Total		Count	29	29	29	87
		% within Parameter3	100.0%	100.0%	100.0%	100.0%

**Table 20:** Chi-square tests

	Value	df	Asymp. significance (2-sided)
Pearson chi-square	5.464^1^	2	0.065
Likelihood ratio	5.392	2	0.067
Linear-by-linear	0.415	1	0.519
Association			
No. of valid cases	87		1

The chi-square table above shows that preference was not significantly dependent on parameters (*P* = 0.065 > 0.05).

1 0 cells (0.0%) have expected count less than 5. The minimum expected count is 6.00.

**Table 21:** Proximate composition of kunu-zaki from composite un-germinated cereal grains (dry matter basis)

Drink type	Moisture content	%Protein	%Fat	%Ash	%Crude fiber	%CHO
SM	39.45	16.3	1.4	1.8	9.8	70.5
AM	49.90	14.82	1.19	2.1	11.0	70.7
AS	43.70	17.7	1.9	1.8	8.0	70.4

The significant test shows that there is no significant difference in the proximate composition of ungerminated cereal blends in composite mix.

**Table 22:** Analysis of variance (ANOVA)

	Sum of squares	df	Mean square	*F*	Sig.
Proximate composition of kunu-zaki					
Between groups	9.224	2	4.612	0.006	0.994
Within groups	11579.115	15	771.941		
Total	11588.339	17			

As the probability value (0.994) is greater than the significant level (0.05), it shows that it is not significant. The significant test shows that there is no significant difference in the proximate composition of ungerminated cereal blends in composite mix.

**Table 23:** Proximate composition of kunu-zaki from composite germinated cereal grains (dry matter basis)

Drink type	Moisture content	%Protein	%Fat	%Ash	%Crude fiber	%CHO
SMG	37.5	17	4.1	1.74	11.3	77.1
AMG	43.92	18.54	0.43	1.2	13.1	79.8
ASG	42.6	13.97	1.33	4	17.73	80.6

The significant test shows that there is no significant difference in the proximate composition of germinated cereal blends in composite mix.

**Table 24:** Analysis of variance

	Sum of squares	df	Mean square	*F*	Significance
Proximate composition of kunu-zaki					
Between groups	11.699	2	5.849	0.007	0.993
Within groups	13365.713	15	891.048		
Total	13377.412	17			

As the probability value (0.993) is greater than the significant level (0.05), it shows not significant. The significant test shows that there is no significant difference in the proximate composition of germinated cereal blends in composite mix.

## Conclusion

The sensory advantage resulting from the inclusion of acha in the processing of kunu-zaki with either sorghum or millet has been demonstrated by this study. An overall consumer preference for the germinated acha–sorghum composite cereal-based kunu-zaki was also exhibited. Preference for germinated acha–millet kunu-zaki followed closely. The study underscores the corresponding values which accompany the use of composite cereal mixes for the processing of kunu-zaki over the use of noncomposite cereals as is the common practice today. Though sprouting of the cereals did not seem to significantly affect proximate composition of kunu-zaki of the composite cereal blends, sprouting did improve the sensory qualities of the resulting drink. This study provides valuable information on the consumer acceptance of new kunu-zaki varieties made from different cereal blends and is helpful for manufacturers who wish to embark on the large-scale commercial production of kunu-zaki beverage.
